# Socioeconomic inequalities in cancer survival in Scotland 1986–2000

**DOI:** 10.1038/sj.bjc.6603980

**Published:** 2007-09-18

**Authors:** L G Shack, B Rachet, D H Brewster, M P Coleman

**Affiliations:** 1 Non-Communicable Disease Epidemiology Unit, London School of Hygiene and Tropical Medicine, Keppel Street, London WC1E 7HT, UK; 2 North West Cancer Intelligence Service, 2nd Floor Muspratt Building, University of Liverpool, Liverpool L69 3GB, UK; 3 Scottish Cancer Registry, NHS National Services Scotland, Gyle Square, 1 South Gyle Crescent, Edinburgh EH12 9EB, UK

**Keywords:** deprivation, socioeconomic inequalities, relative survival, Scotland

## Abstract

We analysed trends in 5-year survival of the 18 commonest cancers in Scotland diagnosed between 1986 and 2000 and followed up to 2004 in each of five deprivation groups based on patients postcode of residence at diagnosis. We estimated relative survival up to 5 years after diagnosis, adjusting for the different background mortality in each deprivation group by age, sex and calendar period. We estimated trends in overall survival and in the deprivation gap in survival up to 2004. Five-year survival improved for all malignancies except bladder cancer and was associated with a widening in the deprivation gap in survival. For 25 of 30 cancer–sex combinations examined, 5-year survival was lower among more deprived patients diagnosed during 1996–2000, and the deprivation gap in survival had widened since 1986–1990 for 15 of these 25 cancers, similar to the trends seen in England and Wales.

Cancer survival is known to vary by socioeconomic level in many parts of the world (Kogevinas and Porta, 1997; Woods *et al*, 2006). In England and Wales, such inequalities have been demonstrated for most adults diagnosed with cancer during 1971–1990 (Coleman *et al*, 1999). Survival improved for adults diagnosed with the 20 most common cancers in England and Wales during the period 1986–1999, but socioeconomic inequalities in survival widened (Coleman *et al*, 2004).

In Scotland, despite substantial recent improvements in survival (Scottish Cancer Intelligence Unit, 2004), cancer mortality has historically been higher, and survival lower, than in England and Wales (Coleman *et al*, 2003; Quinn *et al*, 2005). To the extent that the link between survival and socioeconomic status is causal, the lower survival in Scotland may be partly attributable to a lower average socioeconomic level (Griffiths and Fitzpatrick, 2001). Socioeconomic inequalities in cancer survival in Scotland have been examined, but to our knowledge, trends in these inequalities have not been evaluated. We investigated socioeconomic differences in cancer survival among patients diagnosed in Scotland during 1986–2000, and trends in these inequalities over time.

## MATERIALS AND METHODS

We examined the data for 357 658 adults (aged 15–99 years) diagnosed with a first, invasive, primary, malignant neoplasm (excluding nonmelanoma skin cancer, ICD-10 C00-C97 excluding C44) in Scotland between 1 January 1986 and 31 December 2000 (the most recent year of complete data available at the time) and registered at the Scottish Cancer Registry. Incident cases were linked to death details provided by the General Register Office for Scotland. Data were extracted for analysis on 13 April 2005. The vital status was considered to be known for all patients up to 31 December 2004. Patients identified from a death certificate only were excluded (3.4% of all registrations). About 8.5% of records (31 982) with a second or later tumours were excluded.

Patients were matched to socioeconomic categories based on their postcode of residence at diagnosis, using the 1991 census-derived Carstairs Deprivation Index Score (Carstairs, 1995) for those diagnosed during 1986–1995 and the Scottish Indices of Multiple Deprivation (IMD) 2004 score for those diagnosed during 1996–2000 (General Register Office for Scotland, 2006). Socioeconomic category was based on the geographic level of postcode sector (*n*=4660) for Carstairs and data zone (*n*=770) for IMD. The combined IMD score was used to assign deprivation, rather than only the income domain score, because this was consistent with the Carstairs deprivation group and is the system used by the Scottish Cancer Registry (Measuring Deprivation Subgroup, 2004). The five deprivation categories were derived from quintiles of the national distribution of area deprivation scores in Scotland.

Methods for analysis have previously been published (Coleman *et al*, 2004). Briefly, relative survival at 5 years after diagnosis was estimated for patients diagnosed with cancer and resident in Scotland. Relative survival is the ratio of observed survival of cancer patients and the survival that would have been expected if the patients had had the same age-, deprivation- and sex-specific mortality in each period as the general population (Berkson and Gage, 1950). Period life tables by single year of age (up to 99 years), sex and deprivation category were derived from the numbers of deaths for 1990–1992 and 2000–2002 (General Register Office for Scotland, 2006). Corresponding population denominators were drawn from the 1991 and 2001 census populations from the General Register Office for Scotland (2006).

Relative survival was estimated for each of the 20 most common cancers by deprivation category and sex for each of three periods of diagnosis; 1986–1990, 1991–1995 and 1996–2000. The 1990–1992 life tables were used to estimate background mortality for patients dying in 1986–1995 and the 2000–2002 life tables for those dying in 1996–2004.

We used the maximum likelihood approach for individual records (Estève *et al*, 1990) to estimate relative survival using an algorithm developed for similar analyses (Coleman *et al*, 2004) in STATA software (Statacorp, 2004). Survival probabilities were estimated monthly for the first 6 months, then quarterly up to 1 year, then every 6 months from 1 year to 5 years. Survival for cancer of the larynx was estimated using monthly intervals up to 6 months, then 6 monthly up to 3 years and yearly up to 5 years. The cohort approach was used for patients diagnosed during 1986–1990 and 1991–1995, while 5-year survival was based on the complete approach for those diagnosed in 1996–2000.

**Figure 1:**
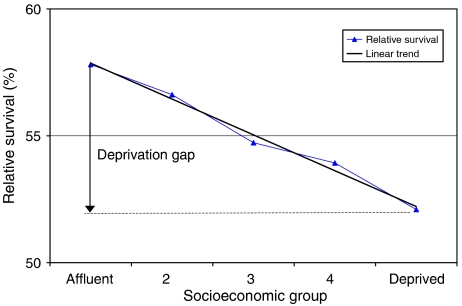
Fitted deprivation gap in 5-year relative survival (%): colon cancer, Scotland, men diagnosed during 1996–2000.

Variance-weighted linear regression (Grizzle *et al*, 1969) was used to estimate temporal change in survival and the survival gradient across deprivation categories. For each time period, the ‘deprivation gap’ in survival was estimated as the absolute fitted difference between 5-year survival in the most deprived and the most affluent categories, estimated from the regression model (Coleman *et al*, 2004; Figure 1). The deprivation gap is described as negative if survival is lower in the most deprived than the most affluent group. Temporal change in the deprivation gap was estimated by inclusion in the model of an interaction term between period of diagnosis and deprivation group.

Survival could not be reliably estimated within deprivation groups for cancers of the pancreas, testis or larynx (women), due to small numbers of cases and/or deaths in some intervals after diagnosis. Survival is high for testicular cancer, and for laryngeal cancer in women, and very few deaths occurred 3–5 years after diagnosis. Most pancreatic cancer patients died within a year of diagnosis.

## RESULTS

Five-year relative survival improved for most malignancies during the period 1986–2000 (Table 1). It increased rapidly, at 7–8% every 5 years, for cancers of the breast (women) and rectum (both sexes), and for all leukaemias combined (both sexes). Five-year survival from prostate cancer rose by an average of 11% every 5 years.

For patients diagnosed during 1996–2000, the deprivation gap in survival was negative (survival lower among the deprived than the affluent) for 25 of the 30 cancer–sex combinations, and statistically significant for 9 of these: colon (both sexes), rectum (women), larynx (men), lung (men), melanoma (women), breast (women), prostate and bladder (men; Table 1).

The deprivation gap in survival was negative for 15 of the 25 cancers diagnosed among patients during 1996–2000, the deprivation gap had widened significantly since 1986. By contrast, the deprivation gap in survival became significantly smaller over the same period for oesophagus (men), stomach (men), brain (men), non-Hodgkin's lymphoma (men), myeloma (women) and leukaemia (men).

Differences in 5-year survival patterns between men and women were observed for all 12 cancers included in the analysis that arise in both sexes. The deprivation gap widened for all these cancers among women, but it was almost stable among men. So, whereas women diagnosed during 1986–1990 experienced a clear survival advantage over men (data not shown), this advantage having disappeared for those diagnosed during 1996–2000.

The deprivation gap widened for uterine cancer, but not for cancers of the breast (women), ovary or cervix. By contrast, the deprivation gap widened by about 3% every 5 years for cancers of the larynx (men) and prostate, leading to large socioeconomic differences in survival by 1996–2000.

Bladder cancer survival decreased over time in both sexes, but women had significantly lower survival than men. The deprivation gap in survival widened by about −4% every 5 years, reaching −7% for patients diagnosed during 1996–2000.

Five-year survival from brain tumours in women was 16% during 1986–1990, but fell from 20% for 1991–1995 to 17% for women diagnosed during 1996–2000. Survival from brain tumours in men improved more in the poor than the rich, so that the deprivation gap had actually reversed (+4% in 1996–2000) with higher survival in the most deprived group.

Survival for non-Hodgkin's lymphoma and myeloma improved by about 5% every 5 years, and leukaemia survival improved even more rapidly, by about 8% every 5 years. No significant socioeconomic difference in survival was seen for any of the haematological malignancies.

## DISCUSSION

For cancer patients diagnosed in Scotland during 1996–2000, 5-year survival was lower among those who lived in more deprived areas. The socioeconomic inequality in survival worsened over the 15-year period 1986–2000, particularly among women.

Socioeconomic differences in cancer survival have been observed in many countries (Kogevinas and Porta, 1997; Ward *et al*, 2004), including the Netherlands (Schrijvers *et al*, 1995a), Canada (Mackillop *et al*, 1997), England and Wales (Coleman *et al*, 1999), Scotland (Scottish Cancer Intelligence Unit, 2000) and the United States (Singh *et al*, 2003). In England and Wales, recent improvements in survival have been more marked in affluent groups, actually widening the socioeconomic inequality in survival (Coleman *et al*, 2004). Our findings are broadly consistent with these studies, both on socioeconomic inequalities in survival and worsening of those inequalities over time. It remains to be determined whether the deprivation gap in survival is widening in other countries.

Further investigation is required, not only to identify the causes of socioeconomic inequality in survival, but also to find out why the inequality is becoming worse, not better.

We assumed linearity when modelling the association between survival and deprivation (five categories), and secular trend in the deprivation gap in survival (three time periods). Flexible functions which allow nonlinear relationships were used to test this assumption (Royston and Ambler, 1998), but no significant departure from linearity was detected.

In a study of breast cancer survival using ecological deprivation indices in England and Wales, the size of geographic unit was found to be more important than the choice of deprivation index (Woods *et al*, 2005). The socioeconomic status assigned to a given postcode sector may also change over time, but a Scottish study found that 80% of postcode sectors in Scotland remained in the same category or only shifted by one socioeconomic group between censuses (Measuring Deprivation Subgroup, 2004).

A similar study carried out in England and Wales was based only on the income domain of the IMD, because health-related factors are a component part (if small) of the overall index (Coleman *et al*, 2004). The complete IMD was recommended for the analysis of Scottish Cancer Registry data, however, because the differential impact was small (Measuring Deprivation Subgroup, 2004).

Possible explanations for socioeconomic variations in survival include variations in comorbidity (Schrijvers *et al*, 1997), stage at diagnosis (Schrijvers *et al*, 1995b; Ionescu *et al*, 1998; Brewster *et al*, 2001) and treatment (Campbell *et al*, 2002; Hole and McArdle, 2002; Jack *et al*, 2006). Only a few studies have adjusted for these factors, or examined their interaction with deprivation. The inequalities in survival have frequently been attributed to more deprived patients presenting at a later clinical stage, but even after adjustment for clinical stage at diagnosis, deprivation differences in survival persist (Schrijvers *et al*, 1995a, 1995b; Campbell *et al*, 2002). Treatment access has also been seen to vary by socioeconomic factors (Madison *et al*, 2004). However, to our knowledge, only one study has evaluated changes over time in deprivation-specific survival and in the deprivation gap (Coleman *et al*, 2004). Temporal changes in prognostic factors that might explain the widening deprivation gap in survival should be investigated, for example, trends in socioeconomic differences in comorbidity, or in access to health care.

Deprivation-specific survival cannot be directly compared between England and Wales and Scotland because the deprivation measures are not defined identically in each country, but comparison of relative changes can be evaluated. Overall, Scotland has higher levels of deprivation than England and Wales (Griffiths and Fitzpatrick, 2001). On average, the deprivation gap in survival for most cancers was larger in Scotland than in England and Wales over the 15-year period 1986–2000 (data not shown). However, the overall picture was very similar, namely a clear deprivation gap in survival which has widened since the late 1980s.

Socioeconomic gradients in colorectal cancer survival may be explained by differences in treatment (Faivre-Finn *et al*, 2002; Guyot *et al*, 2005; Yu *et al*, 2005), stage at diagnosis (Singh *et al*, 2003) or comorbidity (Schrijvers *et al*, 1995a; Wrigley *et al*, 2005), although such differences are not consistent (Ionescu *et al*, 1998; Brewster *et al*, 2001; Wrigley *et al*, 2005). In Scotland, deprived and affluent patients diagnosed during 1991–1994 had similar curative resection rates, but survival was lower among deprived patients, even after adjusting for stage at diagnosis and type of operation (Hole and McArdle, 2002). Stage at diagnosis and treatment are the most important influences on colorectal cancer survival, but their interaction with deprivation remains unclear.

Melanoma of the skin has become increasingly common in the more affluent, particularly women, probably due to an increased ultra-violet exposure. Survival is high in the Nordic countries, but also, uncharacteristically, in Scotland (Coleman *et al*, 2003), probably due to a successful education campaign (MacKie *et al*, 2002) and the increasing proportion of thin tumours (MacKie *et al*, 1997). Despite the high 5-year survival, the deprivation gap in relative survival remains substantial (−6% in men, −4% in women).

The deprivation gap in survival for breast and cervical cancer was 4–5%, but it remained stable throughout the period 1986–2000, as in England and Wales (Coleman *et al*, 2004). Population screening programmes were introduced in Scotland for cervical cancer in 1988 and breast cancer during the 1990s: uptake was higher in more affluent groups, as has been observed in England (Banks *et al*, 2002; Maheswaran *et al*, 2006), the United States (Kothari and Birch, 2004), Canada (Katz and Hofer, 1994) and the Netherlands (Louwman *et al*, 2007). Thus, 81% of affluent women attended the breast-screening programme during 1999–2002, compared to only 58% of deprived women (Information and Statistics Division, 2006). Despite this, the deprivation gap in breast cancer survival among women in the screening age range fell from −3.7% for women diagnosed during 1986–1990 to −0.9% for women diagnosed during 1996–2000 (data not shown).

Prostate cancer incidence and survival increased rapidly since the mid-1990s, both in the United Kingdom (Majeed *et al*, 2000; Pashayan *et al*, 2006a, 2006b) and in other countries (Etzioni *et al*, 2002; Ciatto *et al*, 2005). The trends are partly attributable to increased use of prostate-specific antigen (PSA) testing (Brewster *et al*, 2000), which identifies some cancers that would otherwise have remained asymptomatic and nonlethal (Pashayan *et al*, 2006a, 2006b). The very rapid increase both in survival (11% every 5 years) and in the deprivation gap in survival (reaching −6.9% in 1996–2000) is very similar to the pattern observed in England and Wales (Coleman *et al*, 2004), and appears likely to reflect unequal use of the PSA test within different socioeconomic groups.

Bladder cancer survival increased between 1986–1990 and 1991–1995, but then fell substantially for patients diagnosed during 1996–2000. This appears to be due mainly to a change in the coding of invasive bladder malignancy: in 1995, European recommendations changed to reclassify some papillary urothelial tumours as borderline malignancy (ICD-10 D41.4) or *in situ* (ICD-10 D09.0), rather than invasive. As a result, incidence rates for invasive malignancy of the bladder in Scotland fell by 50% in men and 40% in women between 1996 and 2003 (Information and Statistics Division, 2007). After removal of tumours that had been reclassified as noninvasive, which have a much better prognosis, recorded survival for invasive bladder cancers was lower. The deprivation gap in survival also increased in the late 1990s, reaching −7% by 1996–2000.

## CONCLUSION

Cancer survival in Scotland has improved for all socioeconomic groups, but the increase has been greater for more affluent groups, and socioeconomic inequalities in survival have increased. Possible explanations include widening socioeconomic differences in stage at diagnosis and in access to optimal diagnosis and treatment. The widening socioeconomic gap in cancer survival cannot be attributed to increasing differences in background mortality, since the use of relative survival with deprivation-specific life tables removes these differences in the analysis. In the context of increasing survival, however, socioeconomic differences in comorbidity may also be relevant, particularly if they influence the clinical decision to provide more effective but more aggressive treatment.

## Figures and Tables

**Table 1:** Patterns and trends in 5-year relative survival (%) and the deprivation gap in survival (%), with 95% confidence intervals (CIs): selected cancers, adults (aged 15–99 years) diagnosed in Scotland, 1986–2000

	**Five-year relative survival (%)**						**Deprivation gap in 5-year survival (%)^a^**			
	**Patients diagnosed during 1996–2000**			**Average change (%) every 5 years over the period 1986–2000^b^**			**Patients diagnosed during 1996–2000**		**Average change (%) every 5 years over the period 1986–2000**	
**Malignancy**	**No. of patients**	**Five-year survival (%)**	**95% CI**	**No. of patients**	**Change (%)**	**95% CI**	**Deprivation gap (%)**	**95% CI**	**Change (%)**	**95% CI**
*Oesophagus*										
Men	2109	10.1	8.7–11.6	6302	2.3^c^	1.5–3.2	−0.4	−4.6 to 3.9	1.1^c^	0.5–1.8
Women	1424	9.8	8.2–11.6	4666	0.7	−0.5 to 1.8	−4.6	−9.3 to 0.2	−2.4^c^	−3.2 to −1.5
										
*Stomach*										
Men	2583	15.5	13.9–17.1	9586	2.7^c^	1.4–3.9	1.5	−2.8 to 5.8	0.8^c^	0.1 to 1.4
Women	1768	16.1	14.2–18.1	6479	2.4^c^	1.4–3.5	−2.7	−7.8 to 2.5	−2.6^c^	−3.5 to −1.8
										
*Colon*										
Men	4969	50.8	49.0–52.4	15 409	4.6^c^	3.2–6.0	−5.7^c^	−10.1 to −1.2	−4.4^c^	−5.2 to −3.5
Women	5061	51.0	49.4–52.6	17 216	4.9^c^	3.7–6.1	−6.1^c^	−10.2 to −1.9	−2.4^c^	−3.2 to −1.6
										
*Rectum*										
Men	3190	53.0	50.9–55.1	9506	7.8^c^	6.1–9.5	−5.3	−10.7 to 0.2	−0.7	−1.8 to 0.4
Women	2227	56.0	53.4–58.5	7291	8.1^c^	5.6–10.6	−8.0^c^	−14.5 to −1.5	−2.9^c^	−4.1 to −1.7
										
*Larynx*										
Men	1128	67.3	63.7–70.6	3571	2.9	−0.9 to 6.7	−10.8^c^	−19.9 to −1.8	−3.2^c^	−5.0 to −1.4
										
*Lung*										
Men	12 055	7.1	6.6–7.6	43 414	0.3^c^	0.1–0.6	−1.6^c^	−3.1 to −0.1	−0.6^c^	−0.9 to −0.3
Women	8796	8.1	7.5–8.7	26 707	0.8^c^	0.0–1.7	−1.5	−3.3 to 0.4	−1.2^c^	−1.5 to −0.9
										
*Melanoma*										
Men	1266	85.2	82.5–87.6	3671	4.0^c^	1.3–6.7	−5.9	−12.3 to 0.5	1.3	−0.1 to 2.8
Women	1766	94.6	92.8–95.9	5511	1.4^c^	0.2–2.6	−4.0^c^	−7.6 to −0.5	−1.9^c^	−2.8 to −1.1
										
*Breast*										
Women	16 092	81.6	80.8–82.3	49 910	6.8^c^	5.9–7.7	−4.1^c^	−6.0 to −2.2	−0.2	−0.6 to 0.2
										
*Cervix*										
Women	1654	70.9	68.3–73.2	6265	3.7^c^	1.8–5.5	−4.6	−10.8 to 1.6	0.2	−1.0 to 1.3
										
*Uterus*										
Women	1930	81.2	78.8–83.3	5821	2.9^c^	0.5–5.3	−5.2	−10.7 to 0.3	−4.7^c^	−5.8 to −3.5
										
*Ovary*										
Women	2869	41.1	39.1–43.1	9162	5.3^c^	3.4–7.3	−0.4	−5.6 to 4.8	0.8	−0.2 to 1.7
										
*Prostate*										
Men	9370	72.0	70.7–73.4	26 673	10.8^c^	8.4–12.7	−6.9^c^	−10.3 to −3.4	−2.9^c^	−3.7 to −2.2
										
*Bladder*										
Men	3081	62.6	60.2–64.8	12 139	−1.5	−5.1 to 2.2	−6.7^c^	−12.6 to −0.8	−4.0^c^	−5.1 to −3.0
Women	1451	51.8	48.6–54.9	5521	−2.5	−6.0 to 1.0	−7.3	−15.4 to 0.7	−3.9^c^	−5.3 to −2.4
										
*Kidney*										
Men	1532	44.1	41.2–47.0	4612	2.2	−0.3 to 4.6	−5.2	−12.8 to 2.4	−3.1^c^	−4.6 to −1.5
Women	1066	45.9	42.4–49.3	3240	5.4^c^	3.2–7.6	−4.8	−13.8 to 4.2	0.3	−1.4 to 2.1
										
*Brain*										
Men	942	17.7	15.2–20.3	2945	1.8^c^	−0.1 to 3.7	4.8	−1.8 to 11.4	4.0^c^	2.7–5.3
Women	744	17.3	14.5–20.2	2283	0.9	−1.3 to 3.1	−2.4	−9.4 to 4.5	0.7	−0.6 to 2.1
										
*NHL*										
Men	1846	56.1	53.4–58.7	5710	4.8^c^	1.8–7.8	−5.8	−12.6 to 1.1	1.9^c^	0.5 to 3.2
Women	1972	53.8	51.2–56.4	5961	4.9^c^	3.0–6.9	−2.9	−9.6 to 3.8	−0.5	−1.8 to 0.9
										
*Myeloma*										
Men	674	31.0	26.7–35.2	2268	5.9^c^	3.3–8.4	3.6	−8.6 to 15.7	−0.5	−2.5 to 1.5
Women	704	33.6	29.4–37.8	2329	4.3	−0.2 to 8.8	6.6	−5.2 to 18.4	7.7^c^	5.7–9.8
										
*Leukaemia*										
Men	1473	50.9	47.7–54.0	4554	7.7^c^	5.2–10.1	1.0	−7.1 to 9.0	2.8^c^	1.2–4.4
Women	1253	47.1	43.8–50.3	2787	8.1^c^	5.1–11.0	−1.8	−10.2 to 6.7	−3.0^c^	−4.5 to −1.4

a The deprivation gap shown as negative if fitted survival was lower in the most deprived than the most affluent group. The trend in the deprivation gap is shown as negative if the value became smaller (e.g. 6–4% or −3 to −5%), and positive if the value became larger.

b Adjusted for deprivation (see text).

c Statistically significant at 5%.
